# Neoadjuvant treatment of rectal cancer: where are we now?

**DOI:** 10.1093/gastro/gow017

**Published:** 2016-07-12

**Authors:** Aparna Kalyan, Shaina Rozelle, Al Benson

**Affiliations:** ^1^Developmental Therapeutics Program of Division of Hematology & Oncology, Robert H. Lurie Comprehensive Cancer Center of Northwestern University, Chicago, IL USA; ^2^Division of Hematology & Oncology, Robert H. Lurie Comprehensive Cancer Center of Northwestern University, Chicago, IL USA

**Keywords:** rectal cancer, neoadjuvant chemoradiation, 5-fluorouracil, local recurrence

## Introduction

Colorectal cancer is the third leading cause of cancer-related mortality in the United States [1]. Of this group of patients, approximately 39 000 cases of rectal cancer were reported in the US in 20151]. Treatment of rectal cancer truly requires combinatorial therapy with surgery, chemotherapy and radiation (RT), which now comprise the cornerstone of treatment for rectal cancer. Advanced rectal and colon cancers are generally treated similarly, with most clinical trials not distinguishing between these two anatomic origins. This is in contrast to early stage and locally advanced disease in which the natural histories are distinct, stemming from the fact that the vascular supply for the rectum drains into the inferior vena cava instead of the portal vein [2]. The difference in vascular drainage results in an increase in pulmonary metastases rather than liver metastases [2]. Historically, recurrence within the pelvis has been more common than distant metastases. It is not surprising that treatment objectives focus on minimizing and/or eliminating both local recurrence and distant metastases. Early in the 1970s and 1980s, the recurrence rates were extremely high, nearing 50%, which led to numerous clinical studies evaluating the role of postoperative RT and adjuvant therapy with 5-fluorouracil (5FU) as the backbone. Consensus guidelines from 1990 have established trimodality therapy with chemotherapy, RT and surgery as the standard of care for locally advanced rectal cancer (stage II/stage III) [2,3]. Significant improvements in local disease control have been achieved ever since with the introduction of total mesorectal excision and neoadjuvant chemoradiotherapy (CRT). More recently, questions have been raised as to whether trimodality therapy in the neoadjuvant setting is truly required to obtain disease control for all patients with locally advanced rectal cancer. Furthermore, while local recurrence rates have been stable at 5–6% [4] with this trimodality strategy in recent clinical trials, distal recurrence rates for locally advanced rectal cancers remain at around 25% [5]. In fact, metastases now represent the main cause of death. It is for these reasons that new studies are evaluating the role of systemic chemotherapy in the neoadjuvant setting to address micrometastatic disease and hence potentially reduce the rate of distant recurrence [2,6–8]. In this brief review, we summarize the current literature for neoadjuvant treatment of rectal cancer.

### Staging in rectal cancer

Management of locally advanced rectal cancer is complex, in part due to the necessity of integrating multi-modality treatment consisting of chemotherapy, RT and surgery, which are often required for curative intent. The timing and sequencing of these modalities are challenging because the location of the rectal tumor, the extent of spread and nodal involvement all determine optimal delivery of these treatments. The objective of neoadjuvant treatment remains optimization of disease-free survival (DFS) and overall survival (OS) while minimizing toxicity from RT and chemotherapy and eliminating local recurrence [2].

Early-stage disease, defined as T1-2N0, is usually treated with surgery alone. Locally advanced disease, defined as stage II/III disease, requires initial clinical staging with pelvic MRI and endoscopic rectal ultrasound (ERUS) evaluation to determine the extent of disease and nodal involvement. Staging provides critical information about the likelihood of achieving a complete resection (R0) as well as the likelihood of sparing the rectal sphincter and thereby maintaining fecal continence [2]. Colonoscopic evaluation is required for all patients to determine the extent of resection that will be required and to explore for synchronous lesions. MRI is a vital tool for presurgical management assessment as it can better delineate encroachment on the mesorectal fascia and thereby help determine the potential for a positive radial margin at the time of surgery [8]. The precision of MRI in this setting was evaluated in the MERCURY trial in which high-resolution MRI accurately predicted whether the surgical resection margins were clear or affected by tumor [9].

### Perioperative chemotherapy and radiation

The evolution of total mesorectal excision (TME) has revolutionized the oncological outcomes for patients with resectable rectal cancer. TME uses sharp dissection along the mesorectal fascia (MRF), leading to significantly lower local recurrence rates at 10-year follow-up [10–13].

Historically, trials demonstrated that postoperative RT with concurrent 5FU (used as a radiosensitizer) was an effective strategy for decreasing the rates of local recurrence [14,15]. The German Rectal Cancer Trial randomized 823 patients with cT3–4N+ rectal cancer to either preoperative or postoperative CRT and demonstrated that the rate of local recurrence was lower in the preoperative CRT group than in the postoperative CRT group (6% *vs* 13%; *P* = 0.006). This landmark study defined neoadjuvant CRT as the standard of care [4,16]. Furthermore, toxicity was lower, and quality of life was better in the group that received preoperative therapy. DFS and OS rates were similar between the groups [4,16]. The NSABP R-03 study also supported the advantages of preoperative CRT. This trial demonstrated a statistically significant improvement in 5-year DFS (*P* = 0.011) and a trend toward better OS [17]. However, it should be noted that the NSABP-R03 study allowed 6 additional weeks of neoadjuvant chemotherapy compared with the German Rectal Cancer Trial. Both of these landmark studies therefore support and validate the benefit of neoadjuvant 5FU-based CRT for locally advanced rectal cancer compared with postoperative treatment.

A pooled analysis of 3105 patients receiving neoadjuvant CRT demonstrated that local recurrence rates have decreased to as low as 6% [8]. The neoadjuvant approach has led to consistent tumor down-staging, with 15–27% of patients achieving a pathologically complete response (pCR) defined as no residual cancer found on histological examination of the specimen [8,18]. Further emphasizing the importance of obtaining a pCR, this pooled analysis demonstrated that the 5-year survival rate of 484 patients who achieved pCR after CRT was 83% compared with 66% for those who did not achieve a pCR (*P* < 0.0001). Additionally, the 5-year distal metastases-free survival rate was 89% in the pCR group and 75% in the non-pCR group (*P* < 0.0001) [8]. Expectedly, patients who achieved pCR enjoyed better long-term outcomes with organ preservation, decreased likelihood of developing both local and distant recurrence and improved DFS [19–22].

The MRC-07 study randomized > 1200 patients with operable rectal cancer to receive either preoperative radiotherapy or surgery followed by postoperative CRT (with concurrent 5FU) [23]. The primary endpoint was local recurrence. A 61% reduction in the relative risk of local recurrence was seen in patients receiving preoperative radiotherapy (hazard ratio [HR]: 0.39, *P* < 0.0001), with 6.2% absolute difference at 3 years (95% CI: 5.3–7.1) 23. A further relative improvement in DFS of 24% was seen in patients receiving preoperative radiotherapy (HR: 0.76, *P* = 0.013). The OS was similar for both groups [23].

There are potential scenarios in which preoperative treatment may not necessarily be the best option. These include patients who present with either very small or proximal T2/T3 tumors where chemoradiation may in fact represent over-treatment [2] exposing patients to associated side effects and potential long-term morbidity. Of these patients, who do have nodal involvement or have positive surgical margins, there is evidence from the German Rectal Cancer Trial that postoperative chemoradiation can be safely administered [2]. As previously mentioned, the German trial showed no significant impact on OS; however, chemoradiation was less well-tolerated when administered postoperatively [4].

### Novel concepts in neoadjuvant treatment

A plethora of evidence suggests, with advances in preoperative chemotherapy and surgery, that local recurrence has improved significantly. Distant metastases nonetheless continue to represent a major problem for rectal cancer patients. A pooled analysis of five European randomized controlled trials (RCTs) demonstrated that the 5-year distant metastasis rate was 30.8% in 2759 recruited patients [5]. Furthermore, in a study conducted using the National Comprehensive Cancer Network (NCCN) Colorectal Cancer Database, patients with rectal cancer were evaluated on the frequency of receiving neoadjuvant and postoperative systemic chemotherapy. Results of that study indicated that the number of patients who completed postoperative treatment was significantly lower than anticipated. [24]. From these observations, a shift is emerging towards administering full-dose systemic treatment in the neoadjuvant setting to minimize micro-metastatic disease.

A phase II study evaluated neoadjuvant capecitabine/oxaliplatin (CAPOX) before CRT and surgery in newly diagnosed patients with MRI-defined poor-risk rectal cancer that included tumors with a threatened circumferential resection margin, T3 tumors at or below levators, tumors beyond 5 mm into perirectal fat, T4 tumors and T1-4N2 tumors [25]. This study demonstrated that the radiologic response rate after CAPOX was 88% and increased to 97% at the completion of CRT [25].

**Figure 1.**
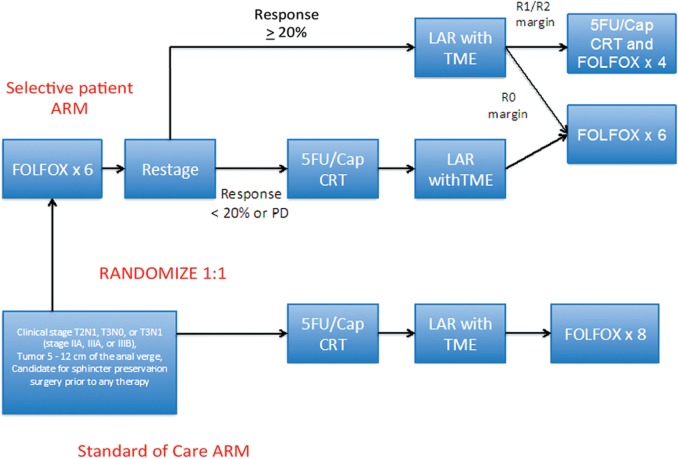
Schema for PROSPECT study. FOLFOX: 5FU/leucovorin + Oxaliplatin; CRT: chemoradiation therapy; LAR: low anterior resection; TME: total mesorectal excision; Cap: Capecitabine The PROSPECT study is evaluating preoperative RT followed by surgery and adjuvant therapy (the current standard of care) versus selective preoperative RT and evaluation before TME. The study aims to reduce the use of pelvic RT in patients who might not benefit from this treatment. All patients who meet criteria will be randomized in a 1:1 fashion.

More recently, a single-center pilot trial from Memorial Sloan Kettering Cancer Center evaluated the concept of selective use of chemoradiation for patients with intermediate risk rectal cancer as determined by MRI. This phase II study enrolled patients with tumors 5–12 cm from the anal verge with no threatened radial margin. Patients received induction FOLFOX-bevacizumab for 6 cycles followed by restaging [26]. Those who had a clinical response from the induction regimen did not receive any further preoperative treatment and proceeded to TME surgery. Patients who did not obtain an adequate response received additional CRT prior to surgery. Of the 32 patients enrolled, 30 patients achieved R0 resection with induction chemotherapy alone. The remaining two patients were intolerant of FOLFOX-bevacizumab, received CRT instead and also subsequently underwent successful R0 resection [26]. This pilot study demonstrated that chemotherapy alone is sufficient for local and distant disease control in carefully selected patients and provided the background to support the currently ongoing PROSPECT study available across the United States. This is a phase II/III randomized trial evaluating the impact of selective use of RT in contrast to standard neoadjuvant CRT for locally advanced rectal cancer. Patients in the intervention arm receive 6 cycles of FOLFOX chemotherapy followed by careful restaging with either pelvic MRI or ERUS. Those patients whose disease is responding (as estimated based on a clinical response ≥ 20 %) proceed directly to rectal cancer resection followed by postoperative systemic therapy at the discretion of the primary provider (Figure 1) (NCT01515787). For those who do not respond, CRT is administered. The study control arm is standard CRT followed by TME surgery and adjuvant chemotherapy.

### FORWARC study

**Figure 2.**
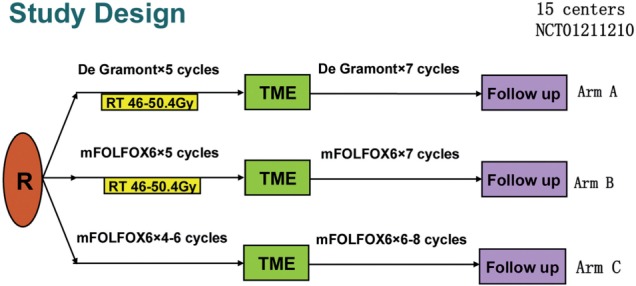
Schema for FORWARC study (adapted from Jianping Wang) Arm A: Traditional CRT followed by surgical resection. Patients then received adjuvant chemotherapy. Chemotherapy was given per the De Gramont regimen consisting of bolus 5FU/LV as well as infusional 5FU. Arm B: Neoadjuvant mFOLFOX with combined RT therapy followed by surgical resection. Adjuvant chemotherapy consisted of mFOLFOX. Arm C: Neoadjuvant chemotherapy with mFOLFOX followed by surgery and adjuvant therapy.

Preliminary results from the large Chinese multicenter FORWARC study were presented at the annual American Society of Clinical Oncology (ASCO) Scientific meeting in 2015. This study investigated whether perioperative mFOLFOX6 chemotherapy improves DFS in locally advanced rectal cancer. Patients with clinical stage II-III rectal cancer within 12cm of the anal verge were randomized to receive 5FU with RT (control arm) or mFOLFOX6 with RT (FOLFOX-RT arm) or 4–6 cycles of mFOLFOX6 alone (FOLFOX arm) (Figure 2) [27]. Additional postoperative RT was allowed if required. Preliminary results demonstrated that the R0 resection rates were 90.1% (control arm), 88.2% (FOLFOX-RT arm) and 91.2% (FOLFOX arm). The pCR rate was significantly higher in the FOLFOX RT arm (31.3%) compared with the control arm (12.5%) and FOLFOX arm (7.4%) (*P* = 0.001). The overall down-staging was similar across all arms. As expected, greater toxicity and postoperative complications were observed in patients who received RT. Based on these preliminary data, it appears that mFOLFOX6 concurrent with RT resulted in a higher pCR rate and that neoadjuvant mFOLFOX6 alone achieved similar down-staging with less toxicity and postoperative complications compared with preoperative 5FU with RT [27].

## Conclusion

While significant advancements in the management of locally advanced rectal cancer have occurred over the last 30 years—resulting in improved local control rates in particular—the risk of distant metastases remains an ongoing problem. New novel strategies to select patients for either CRT or upfront neoadjuvant chemotherapy appear to be promising and should result in decreased morbidity for some patients. There may also be patients with a pCR after neoadjuvant therapy who may be candidates for a “watch-and-wait” approach—thus avoiding surgery—which is the subject of current clinical investigation [28]. A new US NRG trial is planned to explore the use of other radiosensitizing agents in the neoadjuvant setting. Clearly, new systemic approaches, perhaps based on genomic profiling, will be needed to reduce the risk of metastatic disease.

*Conflict of interest statement:* none declared.
